# Quinine and Blackwater Fever

**Published:** 1903-05-23

**Authors:** 


### QUININE AND BLACKWATER FEVER.

A case of considerable importance in view of its bearing on the pathology of blackwater fever is reported by W. G. Ross, M.D., and G. C. Low, M.B.[1] An Englishman, aged 25, went to the Gold Coast in 1901. He had four not very severe attacks of fever between January 1902 and September of that year. During the periods of fever and at irregular intervals between he was treated with quinine, which he took in 10-gr. doses. In October, thinking that he was about to have an attack of malaria, he took 10 grs. of quinine, and this was followed by blackwater fever. The quinine was taken at 5 p.m., and at about midnight he had vomiting, diarrhœa, and frequent micturition. He noticed his urine was black. This attack kept him in bed for three days, the blackwater having passed off in 24 hours. At the same time he became deeply jaundiced. No further attacks ensued while the patient remained in Africa, and he arrived home last March. On the 11th of that month being out of sorts he took 10 grs. of quinine ; this dose was followed by vomiting, diarrhœa, and the passage of blackwater. Subsequently, while under observation in the Seamen's Hospital at Greenwich, his idiosyncrasy towards quinine was finally settled, for 10 grs. of quinine again caused hæmoglobinuria. Examination of the urine showed it to contain albumen, methæmoglobin and a few hæmoglobin casts. The importance of the recognition of such a reaction to quinine on the part of anyone lies in the fact that such a person is debarred from residence in any quarter where malaria is rife.

[1] Journal of Tropical Medicine, May 1.

